# Nutrition With Skimmed Breast Milk in an Infant With Long Chain 3‐Hydroxyacyl‐coA Dehydrogenase Deficiency

**DOI:** 10.1002/jmd2.70018

**Published:** 2025-05-07

**Authors:** Clara Alonso‐Diaz, Diana Escuder‐Vieco, Pilar Quijada‐Fraile, Delia Barrio‐Carreras, Patricia Pérez‐Mohand, Elena Martín‐Hernández, Carmen Rosa Pallas‐Alonso, Nadia Raquel García‐Lara

**Affiliations:** ^1^ Neonatology Department Research Institut i+12. 12 de Octubre Hospital. Universidad Complutense Madrid Spain; ^2^ Aladina‐MGU Regional Mother's Milk Bank Research Institut i+12. 12 de Octubre Hospital Madrid Spain; ^3^ Enfermedades Mitocondriales‐Metabólicas Hereditarias Research Institut i+12. 12 de Octubre Hospital Madrid Spain

**Keywords:** (skimmed) breast milk feeding, enteral nutrition, human milk, LCHADD, long‐chain fatty acid oxidation defects (LC‐FAODs), necrotizing enterocolitis, skimmed breast milk

## Abstract

The current standard diet for long‐chain 3‐hydroxyacyl‐CoA dehydrogenase deficiency (LCHADD) in the first months of life includes a special formula low in long‐chain triglycerides (LCT) and enriched in medium‐chain triglycerides (MCT). It involves the interruption of breastfeeding, withholding its nutritional and nonnutritional benefits. We describe the clinical case of a late preterm with 36 weeks gestational age diagnosed with LCHADD through newborn screening (NBS) who developed necrotizing enterocolitis (NEC) and sepsis due to *Escherichia coli* ( *E. coli* ) at 7 days of life. During hospital admission, the patient was fed skimmed breast milk supplemented with MCT oil and a low‐fat MCT‐enriched formula. Because the family wished to continue pumping milk after discharge, they were trained to defat milk using a non‐refrigerated benchtop centrifuge. At home, a similar feeding regime was followed for 4 months. Hospital and home‐produced skimmed breast milk met the dietary treatment requirement of < 1.0 g/dL of fat content. Growth and development during the first 5 months of life were normal, with an improved serum acylcarnitine profile and no decompensation. In this report, we demonstrated that breast milk defatting is a safe and feasible option for patients with LCHADD during hospital admission and at home, providing the benefits of human milk in these patients. This approach could influence dietary management guidelines for metabolic disorders or expand breast milk feeding options for medically complex infants.

**Summary**

- Skimmed breast milk in patients with long‐chain 3‐hydroxyacyl‐CoA dehydrogenase deficiency is a safe and feasible option for families who wish to breastfeed during hospital admission and at home.

## 1 Introduction

Long‐chain 3‐hydroxyacyl‐CoA dehydrogenase (LCHAD) deficiency (LCHADD) is an inborn error in fatty acid metabolism that results in insufficient energy production and accumulation of fatty acid intermediates. LCHADD and trifunctional protein (TFP) deficiency (TFPD) are due to the impairment of mitochondrial TFP [1]. The TFP enzyme activities include long‐chain enoyl‐CoA hydratase, LCHAD, and 3‐ketoacyl‐CoA thiolase. Mutations in the HADHA gene usually cause LCHADD [2]. The blood acylcarnitine profile does not distinguish LCHADD from TFPD; long‐chain hydroxyl acylcarnitines are elevated in both [3].

Isolated LCHADD may present with a severe‐to‐intermediate phenotype. Neonates with a severe phenotype present in the first days of life with hypoglycemia, lactic acidosis, hepatomegaly, encephalopathy, and often cardiomyopathy [1].

These diseases can be diagnosed early through newborn screening (NBS). In Spain, the NBS of LCHADD has been mandatory since 2013 [4]. However, in the Madrid region, it was initiated in 2011 [5, 6].

The primary goal of dietary management of all long‐chain fatty acid oxidation disorders (LC‐FAODs) is to primarily avoid catabolic situations with frequent feeding and to limit long‐chain triglycerides (LCT) and supplement medium‐chain triglycerides (MCT) [2]. The level of fat restriction depends on the genetic mutation and the severity of the disease [7]. In infancy, total fat intake from all sources provides 40%–45% of energy, and at least 10% of dietary calories should come from LCT to maintain normal levels of essential fatty acids [2]. Docosahexaenoic acid (DHA) supplementation is also recommended [2, 3].

As breast milk has a high fat content, breastfeeding may need to be discontinued according to the consensus of the dietary recommendations for LCHADD. Therefore, for newborn patients, a special infant formula low in LCT and high in MCT is recommended [8].

It needs to be stated that breast milk feeding confers unique nutritional and non‐nutritional benefits to infant and mother [9, 10], whereas the treatment of LCHADD with formula does not. This is why an alternative food approach is needed.

Skimmed breast milk (SBM) is the nearly fat‐free fraction of breast milk [11]. SBM is routinely used to treat chylothorax in infants, providing the human‐specific nutritional and immune benefits of breast milk [11, 12]. This practice of defatting breast milk has been previously described in other LC‐FAOD cases [13, 14].

We present a case of a patient with LCHADD who was fed supplemented SBM during hospital admission and at home until 4 months of age. He showed adequate growth and development and had no complications. We believe reporting this case is relevant for providing sufficient information and encouraging experts to include this option in their dietary guidelines.

## 2 Case Report

We present the case of a late preterm neonate (male) with 36 weeks gestational age. Nonconsanguineous parents. He was the firstborn of this mother. The pregnancy was controlled, and intrauterine growth retardation (IGR) stage 1 was diagnosed. After a vaginal delivery, the patient required resuscitation with intermittent positive‐pressure ventilation. Apgar test was 8/9. Birth weight was 1590 g (percentile 1), length was 42 cm (percentile 2), and head circumference was 31 cm (percentile 11).

In the first 5 days of life, the patient presented with hypoglycemia (19 mg/dL at 2 h) that required intravenous glucose and enteral feeding with breast milk and preterm formula. At 6 days of life, he was transferred to our center after a positive NBS result for LCHADD. After extracting confirmation samples, feeding with a low‐fat MCT‐enriched formula (Monogen) was initiated.

At 7 days of life, the patient presented with fever, metabolic acidosis, hyperlactacidemia, and abnormal findings on abdominal examination. Abdominal radiography and ultrasonography findings were compatible with those of necrotizing enterocolitis (NEC). An exploratory laparotomy revealed pneumatosis; however, intestinal resection was unnecessary.

*Escherichia coli* was detected in the blood cultures. Meningeal involvement was ruled out by cerebrospinal fluid culture. He received gentamicin treatment for 9 days.

The patient required conventional mechanical ventilation for 4 days, vasoactive support (for 36 h), and several blood product transfusions because of anemia, thrombocytopenia, and coagulopathy. The echocardiographic results were normal.

During NEC episode, parenteral nutrition (without lipids) was given for 8 days to prevent decompensation. A high intravenous glucose (13.9 mg/kg/min) was required to achieve the total calories. At 12 days of life, trophic feeding with SBM was initiated. Owing to insufficient breast milk, SBM was combined with a low‐fat formula enriched with MCT (Monogen) at 18 days of life. Exclusive enteral nutrition was achieved at 21 days of life. The mother was instructed to keep up milk production by adequate lactation consultation and pumping management.

Elevated levels of C16‐OH and C18‐OH were detected in the serum acylcarnitine profiles (Table 1). These results suggest an LCHADD similar to that of NBS. Molecular studies detected the pathogenic variant c.1528G>C (p.Glu510Gln) with homozygosity in HADHA, confirming the diagnosis of LCHADD.

**TABLE 1:** Evolution of acylcarnitine profile.

Age	C16‐OH (μmol/L) (NV: 0.00–0.03)	C18‐OH (μmol/L) (NV: 0.00–0.01)	C16‐OH + C18‐OH (μmol/L) (NV: 0–0.04)	Free carnitine (μmol/L) (NV: 19.4 ± 7.5)	Total AC (μmol/L)	Esterified carnitine (μmol/L)
6 days (pre intervention)	0.37	0.35	0.72	31.19	49.81	18.62
3 months (post intervention)	0.21	0.16	0.37	41.6	50.76	9.16

Abbreviations: AC, acylcarnitines; NV, normal values.

During hospital admission, starting at Day 12 of the child's life, milk defatting was performed in a refrigerated centrifuge (Eppendorf, 5910Ri) at 3000 rpm, 15 min, 2°C. These conditions were the same as those used in the unit for defatting milk in patients with chylothorax. The nutritional content of milk before and after centrifugation was analyzed using a milk analyzer (MilkoScan FT2, FOSS). Hospital defatted breast milk contains between 0.12 g and 0.58 g/dL of lipids (Table 2). The quantity of lipids in the defatted breast milk was modified according to the patient's needs without exceeding 10% of the dietary calories.

**TABLE 2:** Nutritional content of skimmed milk.

Nutritional content of the milk centrifuged in the hospital				
Days of life	Energy (Kcal/dL)	Fat (g/dL)	Protein (g/dL)	Lactose (g/dL)
13	42.32	0.12	1.95	8.36
16		0.47	1.8	8.45
17		0.22	1.73	8.58
20		0.25	1.61	8.75
23		0.32	1.56	8.74
25		0.35	1.47	8.82
27		0.32	1.48	8.70
30	45.15	0.51	1.48	8.66
32		0.5	1.49	8.72
34		0.55	1.5	8.62
37		0.58	1.5	8.64
39		0.57	1.49	8.63

Nutritional content of the milk centrifuged at home			
Daily samples	Fat (g/dL)	Protein (g/dL)	Lactose (g/dL)
1	0.51	1.47	8.52
2	0.43	1.49	8.59
3	0.51	1.48	8.57
4	0.86	1.47	8.59
5	0.56	1.49	8.71
6	0.24	1.50	8.89
7	0.88	1.42	8.48
8	0.55	1.42	8.53
9	0.36	1.45	8.86
10	0.20	1.44	8.73
11	0.39	1.43	8.57

During admission, the patient received a maximum of 112 mL/kg/day of SBM, supplemented with a low‐fat formula enriched with MCT (Monogen) and MCT oil until the desired caloric intake was achieved (Table 3). He also received DHA supplementation (60 mg/day).

**TABLE 3:** Nutritional content of the enteral feeding.

Days of life	Weight (kg)	Special formula (mL/day)	Skimmed milk (mL/day)	MCT oil (mL/day)	Enteral energy (kg/day)	Enteral long‐chain fats (% of total calories)	Enteral of medium‐chain fats (% of total calories)	Enteral proteins (g/kg/day)
14	1920	0	32		5			
16	1920	0	134		30			
17	1920	0	215		47			
18	2000	120	120		61	4.3	12.6	2.1
19	2000	160	160		81	4.3	12.6	2.8
20	2000	180	180		92	4.6	12.6	3.0
26	2060	174	200		91	5.6	11.8	2.9
31	2140	220	220		108	6.7	12.2	3.3
32	2200	240	240		114	6.6	12.2	3.5
33	2200	300	180		118	5.9	14.8	3.6
34	2200	300	210		124	6.5	14.0	3.8
37	2320	300	210	3	129	6.1	21.5	3.6

*Note:* During Days 14–17, caloric intake was completed with parenteral nutrition (without lipids). During Days 18–22, caloric intake was completed with only intravenous glucose (1.3–3.3 mg/kg/min). Special formula: Monogen of Nutricia Metabolics.

The family wished to continue pumping breast milk at home. Consequently, they were trained in defatting breast milk using a nonrefrigerated benchtop centrifuge (Mmoonant 800‐1) with a 110 V motor and a capacity to process up to 135 mL per cycle. It was available online for 115 euros. A food technologist of the milk bank trained the family for 2 days with the following protocol:

1. The milk to be centrifuged needs to be placed into BPA‐and phthalate‐free polypropylene tubes that can be heated up to 140°C and are reusable for food use with a pressure cap.
2. The milk level should be the same in all tubes to avoid imbalances in the centrifuge.
3. The milk should be centrifuged for at least 5 min at 3000 rpm. Pretests were carried out at 1500 rpm and 2500 rpm, and it was found that this was not a sufficient speed to be able to separate the fat layer from the milk completely.
4. Subsequently, the tubes have to be kept at freezing temperatures (approximately −20°C) for 15–30 min until the fat layer on top is sufficiently hard. Since the centrifuge does not have a cooling system, the fat layer needs to be further cooled to a consistency hard enough to be removed with a spatula without mixing with the rest of the non‐fat milk.
5. Finally, the fat layer is separated using a stainless‐steel spatula, leaving the defatted milk in the tube.

**FIGURE 1:**
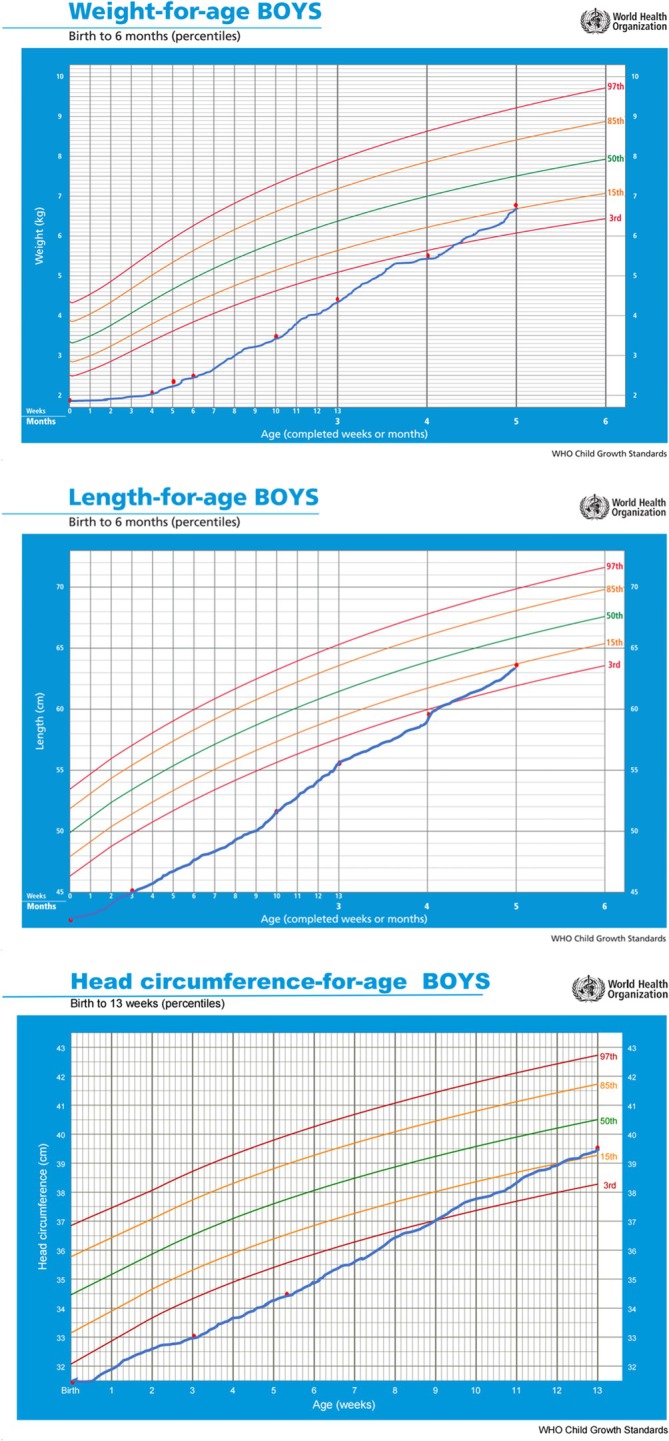
Growth graphs according to postmenstrual age (weight, length, and cephalic perimeter).

The amount of fat obtained by centrifugation at home was analyzed (MilkoScan FT2, FOSS) (Table 2) and was found to be safe for the patient (0.2–0.88 g/dL of lipids) with results similar to hospital defatting and with little variability. This procedure allowed the parents to continue partial SBM feeding by bottle after hospital discharge at 40 days of life. At that moment, the patient received 2–3 feedings per day of SBM and the rest of the special formula (Monogen). He was fed supplemented SBM for 4 months. At 3 months of age, the acylcarnitine profile of the serum improved, showing a decrease in the levels of C16‐OH, C18‐OH, and esterified carnitine. His growth and development were adequate despite early complications, prematurity, and low birth weight. The patient reached the third percentile for weight and length at 3 and 2 months corrected age, respectively (Figure 1). He was admitted to the hospital three times during this period because of mild digestive intolerance, mild acute bronchiolitis, and vaccine reaction. The patient had no acute decompensation related to metabolic diseases.

Neurological development, echocardiography, and abdominal ultrasonography findings at 6 months were normal. No deficiencies in essential fatty acids were observed in erythrocytes.

## 3 Discussion

Here, we present a case of LCHADD in a late preterm neonate with an episode of NEC and *E. coli* sepsis. After recovering from this episode, the patient was successfully fed SBM during hospital admission and at home until the age of 4 months. For milk defatting, we used the same protocol as that used for patients with chylothorax. Because the family wished to continue expressing breast milk, the protocol was adapted, and the parents were trained to defat milk at home. This allowed the infant to benefit from partial breast milk feeding for 4 months when the mother's milk production ceased.

The use of SBM has been previously described in two cases with LC‐FAODs.

Metzler et al. [13] reported a case of NEC in a preterm neonate with LCHADD, who was fed SBM at the hospital (no information about nutrition at home). Kritzer et al. [14] described the use of SBM at home for 12 months in a patient with carnitine acylcarnitine translocase deficiency using a different defatting method (cream separator). Our study shows that it is possible and safe to use SBM in the hospital and at home in neonates with LCHADD. This enhances the evidence to influence dietary management guidelines for metabolic disorders.

Three methods of skimming milk (refrigerated centrifuge, cream separator, and manual separation after refrigeration) were compared in a study [15]. It showed a similar macronutrient composition of SBM defatted by a cream separator and centrifuge. Both methods removed almost all fat and substantially more fat than the manual fat removal method after refrigeration [15, 16]. Other methods for defatting milk at home in patients with chylothorax have also been described [17]. In our patient, we decided to use a portable centrifuge that can be used at home with an efficiency similar to that of a cream separator [18].

NEC as first clinical presentation is not common in LCHADD but has been previously described [13, 19]. Long‐chain fatty acid oxidation may play an important role during early human development, and it has been suggested that normal function of the TFP complex is needed for normal intestinal development and function [20]. Severe NEC observed in these patients may be related to the enzymatic deficiency in these organs during crucial stages of development. Although our patient had other risk factors for NEC, such as premature birth, IGR, and low birth weight, the interruption of breastfeeding may have contributed to NEC development. Therefore, in metabolic disorders that present with intestinal risk, feeding SBM as a preventive measure should be considered. In contrast, MCT‐based formulas (the current standard treatment) increase intestinal risk and do not provide the specific nutritional and immune benefits of breast milk.

Studies on breastfeeding for metabolic diseases are limited. A survey [21] conducted in the United States and Canada on breastfeeding practices for infants with inherited metabolic disorders showed that breastfeeding is less likely to be used for fatty acid oxidation disorders, and the use of measured expressed breast milk is preferred. This can be explained by the high fat content in breast milk and the high risk of decompensations in LCFAODs.

One study in Austria [22] described three preterm infants that were diagnosed with LCHADD by NBS: two of them were fed expressed breast milk, and the other was breastfed on demand. They were not metabolically decompensated at the time of diagnosis and do not describe problems during the breastfeeding period (2–5 months). Although initiating breastfeeding on demand is possible in asymptomatic patients, as proposed by the Austrian group, until new studies are available, defatting milk offers more security and is a good alternative to special formula in patients with symptomatic LC‐FAODs. Also, the addition of oils like MCT or triheptanoin might seem easier, as it is added into bottle feeds, whereas in breastfed babies the addition has to be done additionally. Limitations of defatting milk include that it is not feasible for all families (financial cost, time requirements, training) and long‐term breastfeeding is more difficult, although many of these problems can be reduced with professional support.

In addition, families of children with LC‐FAOD have a high burden of disease owing to the risk of metabolic decompensation and the possibility of irreversible complications [23]. For this reason, healthcare professionals should support families and help them if they wish to maintain expressing breast milk because it empowers parents in the care of their children and provides the benefits of human milk for their infants.

Like formulas for infants with LC‐FAODs, the fortification and supplementation must replace the fat‐soluble vitamins and essential fatty acids that have been lost with the removal of breast milk fat [12].

Potential biases of the study are that it is a single case report and the use of SBM was only for 4 months supplemented with a low‐fat MCT‐enriched formula; therefore, the results cannot be generalized and it does not respond to longer‐term use.

In summary, nutrition with SBM in patients with LCHADD is a safe and feasible option for families who wish to breastfeed during hospital admission and at home. Defatting breast milk at home using a portable centrifuge is another method that should be considered. Healthcare professionals should offer these families all the possible tools to allow SBM feeding with nutritional and immunological benefits.

## Author Contributions

Clara Alonso‐Diaz, Diana Escuder‐Vieco, Pilar Quijada‐Fraile, Delia Barrio‐Carreras, Patricia Perez‐Mohand, Elena Martin‐Hernandez, Carmen Rosa Pallas‐Alonso, and Nadia Raquel Garcia‐Lara contributed to planning, conduct, and reporting of the work described in the article. All authors contributed to the design, implementation, and execution of this study, as well as the writing and editing of the manuscript.

## Ethics Statement

All procedures followed were in accordance with the ethical standards of the responsible committee on human experimentation (institutional and national) and with the Helsinki Declaration of 1975, as revised in 2000. No patient identifiers are included in the article.

## Consent

The family consents to the publication of their child's case (signed consent attached).

## Conflicts of Interest

The authors declare no conflicts of interest.

## Data Availability

The data that support the findings of this study are available from the corresponding author, Clara Alonso‐Diaz, upon reasonable request.
